# In vivo and in vitro study of co-expression of LMP1 and Cripto-1 in nasopharyngeal carcinoma^☆^

**DOI:** 10.1016/j.bjorl.2019.04.002

**Published:** 2019-05-20

**Authors:** Qing Ye, Jing Li, Xiaoyan Wang, Xianzeng Zhang, Jun Lin, Yuting Huo, Zhengzhen Sun, Shusen Xie, Zheng Huang

**Affiliations:** a Fujian Medical University, Fujian Provincial Hospital and Provincial Clinical College, Department of Otolaryngology, Fujian, China; b Fujian Normal University, OptoElectronic Science and Technology for Medicine, MOE Key Laboratory, Fujian, China

**Keywords:** Nasopharyngeal carcinoma, LMP1, Cripto-1, Metastasis, Carcinoma nasofaríngeo, LMP1, Cripto-1, Metástase

## Abstract

**Introduction:**

Nasopharyngeal carcinoma, an epithelial-derived malignant tumor which because of its anatomical location and atypical early symptoms, when diagnosed invasion and metastasis often have occurred. This requires a better understanding of the development mechanism, identifying diagnostic markers, and developing new treatment strategies.

**Objective:**

To study the relationship of LMP1 and Cripto-1 in nasopharyngeal carcinoma.

**Methods:**

The expression of LMP1 and Cripto-1 in specimens obtained from nasopharyngeal carcinoma patients (*n* = 42) and nasopharyngitis patients (*n* = 22) were examined. The expression of LMP1 and Cripto-1 in LMP1-negative and LMP1-positive (CNE1-LMP1) cells were also examined.

**Results:**

The expression of LMP1 and Cripto-1 was significantly higher in nasopharyngeal carcinoma than in nasopharyngitis (*p* < 0.05). Their expression in nasopharyngeal carcinoma with metastasis were significantly higher than that without metastasis (*p* < 0.05), which was correlated with TNM staging (*p* < 0.05). High Cripto-1 expression and high proliferation rate were seen in CNE1-LMP1 cells.

**Conclusions:**

The expression of LMP1 and Cripto-1 in nasopharyngeal carcinoma is positively related. Their co-expression might contribute to the proliferation and metastasis of nasopharyngeal carcinoma.

## Resumo

**Introdução:**

O carcinoma nasofaríngeo é um tumor maligno derivado do epitélio de localização anatômica recôndita e sintomas iniciais atípicos; quando diagnosticado, frequentemente invasão e metástases já ocorreram. Isso requer uma melhor compreensão do seu mecanismo de desenvolvimento, identificação dos marcadores diagnósticos e desenvolvimento de novas estratégias de tratamento.

**Objetivo:**

Estudar a relação de LMP1 e Cripto-1 no carcinoma nasofaríngeo.

**Método:**

A expressão de LMP1 e Cripto-1 em espécimes obtidos de pacientes com carcinoma de nasofaringe (n = 42) e pacientes com nasofaringite (n = 22) foi analisada. A expressão de LMP1 e Cripto-1 em células LMP1-negativas e LMP1-positivas (CNE1-LMP1) também foi analisada.

**Resultados:**

A expressão de LMP1 e Cripto-1 foi significantemente maior na presença de carcinoma nasofaríngeo do que na nasofaringite (*p* < 0,05). Sua expressão em carcinomas com metástase foi significantemente maior do que em casos sem metástase (*p* < 0,05), o que se correlacionou com o estadiamento TNM (*p* < 0,05). Uma alta expressão de Cripto-1 e alta taxa de proliferação foram observadas nas células CNE1-LMP1.

**Conclusões:**

A expressão de LMP1 e Cripto-1 é positivamente relacionada com carcinoma nasofaríngeo. Sua coexpressão pode ser atribuída à proliferação e metástase do tumor.

## Introduction

Nasopharyngeal carcinoma (NPC), an epithelial-derived malignant tumor with special regional, ethnic and family clustering features, is one of the most common cancers in Southeast Asia and South China. Its incidence rate in South China is approximately 200 million.^1^, ^2^ If diagnosed and treated early, 90% survival rate can be achieved. Unfortunately, most of the cases are diagnosed in the late stages and the survival rate is less than 50%. Mainly due to the protection of its anatomical location and atypical early symptoms when diagnosed, invasion and metastasis often occur. Although precision radiotherapy and target chemotherapy have significantly improved the survival rate, more than 30% of patients suffer from local recurrence or distant metastasis and the recurrence rate may be as high as 82%. Once relapsed, the survival rate can be significantly decreased and the median survival time is only 7.2–22 months. The majority of patients died of distant metastasis, the main reason for treatment failure.^3^, ^4^, ^5^, ^6^ Hence, early detection, diagnosis and treatment have a great significance for the improvement of survival rate. This requires a better understanding of the development mechanism, identifying diagnostic markers, and developing new treatment strategies.

Latent membrane protein 1 (LMP1) is an integrated membrane protein that shares the signal transduction pathway with TNF receptor super family members. As a classical oncogene, LMP1 is tumorigenic. In epithelial cells, LMP1 promotes cell growth, stimulates migration and invasion, and regulates squamous epithelial differentiation. The latter effect also can enhance the ability of tumor cell invasion.^7^ LMP1 is the most important oncoprotein expressed by Epstein–Barr Viral (EBV) in NPC cells and essential for the growth and transformation of EBV-mediated infected cells, which play an important role in the occurrence, development, invasion and metastasis of NPC.^8^

LMP1 contains three domains: a short cytoplasmic N-terminus, six transmembrane spanning regions and a large cytoplasmic C-terminal tail. It interacts through the cytoplasmic domains (C-Terminal Activation Regions – CTAR 1 and 2) with Tumor necrosis Receptor Family adapter molecules (TRAFs). Its special structure makes its function similar to TNF Receptor (TNFR) family member CD40 and could trigger a series of cellular signaling pathways with a ligand-independent manner, including NF-κB, JAK/STAT, p38/MAPK, PI3K/Akt and ERK-MAPK/JNK/SAPK. Therefore, it is involved in cancer cell invasion, metastasis and malignant transformation.^9^, ^10^, ^11^, ^12^, ^13^

Cripto-1, also known as teratocarcinogen-derived growth factor-1, is a member of the Epidermal Growth Factor (EGF) Cripto-1-FRL-1-Cryptic (CFC) family. It is a multifunctional modulator involved in embryogenesis and tumor formation and essential in regulating early embryogenesis, promoting cell migration, angiogenesis and stem cell maintenance. Cripto-1 mRNA and/or protein expression has been found in breast, cervix, testis, lungs, colon, stomach, pancreas and ovaries.^14^, ^15^ Numerous studies show that malignant transformation, invasion, metastasis and poor prognosis of tumor are associated with high expression of Cripto-1.^16^, ^17^ Experts suggest that Cripto-1 up-regulation might play a role in the malignant progression and metastasis of human NPC.^18^, ^19^ Cripto-1 protein has been considered as a potential biomarker for the progress of tumor invasion and metastasis.

EBV infection is closely associated with NPC in its main molecular mechanism. However, how LMPl related tumorigenic mechanisms function in NPC have not yet been fully elucidated. Nevertheless, it is known that LMP1 is involved in three classic mitogen-activated protein kinases (MAPK): ERK-MAPK, p38 MAPK and JNK/SAPK via both typical and atypical NF-kappa B pathway and PI3K pathway.^20^, ^21^, ^22^ Cripto-1 plays an important role in embryonic development and tumor progression as a co-receptor of TGF-β related morphological factor Nodal.^23^ Cripto-1 can interact with ras, p38 MAPK and Wnt/B-catenin via the TGF-β signaling pathway and directly regulate classical EMT pathway to promote transcription factors such as Snail, Twist and Slug transcription. Cripto-1 also interacts with TGF-β1 and activin A and B to interfere and weaken their signaling in a variety of cell lines, acts as Glycosyl Phosphatidyl Inositol (GPI) ligand that activates MAPK and PI3K/protein kinase B (Akt) signaling pathways to regulate cell proliferation, migration and survival.^24^, ^25^, ^26^, ^27^ Therefore, it is reasonable to suggest that there is a correlation between LMP1 and Cripto-1 in human NPC.

In this study, immunohistochemistry, western blot and RT-PCR assays were used to investigate the co-expression of LMP1 protein and Cripto-1 protein in the occurrence and development of human NPC. We reported for the first time the correlation of LMP1 and Cripto-1 in human NPC. Our findings may also provide new insights into understanding the molecular mechanism involved in NPC carcinogenesis and progression.

## Methods

### Specimen collection and classification

During February 2011 and February 2016 nasopharyngeal mucosa specimens were collected from 42 patients who suffered from nasopharyngeal carcinoma. Those with MRI confirmed neck lymph node metastasis were identified. All specimens were diagnosed pathologically according to WHO histological classification of nasopharyngeal carcinoma. None of them had received radiotherapy, chemotherapy and other treatment previously. The clinical TNM stage was classified according to the 2002 Union for International Cancer Control (UICC) Head and Neck Cancer Clinical Stage (Version 6) for the nasopharyngeal carcinoma staging. As for the control, at the same period of time tissue specimens were also collected from 22 patients who suffered from nasopharyngitis. All samples were obtained with the patient's informed consent and approved by the Ethics Committee (n° K2016-01-01).

### Immunohistochemistry examination of tissue specimen

Fresh specimens were fixed in 10% neutral formalin and underwent the routine tissue processing steps. Series of rehydrated sections of 2.5 μm thickness underwent the antigen retrieval step of boiling citrate buffer for 1.5 min, then in 3% H_2_O_2_ for 10 min to block the endogenous catalase, followed by PBS rinsing. Elivision 2 Step immunohistochemistry staining method was carried out by first incubating the sections with the corresponding primary antibody, i.e. mouse anti-LMP1monoclonal antibody (1:500) (M0897, DAKO, Denmark) or rabbit polyclonal anti-Cripto-1 antibody (1:60) (ab19917, Abcam, USA), at 37 °C for 1 h, then with goat anti-rabbit or anti-mouse IgG polyclonalantibody H&L (1:5000) (ab6721 and ab6789, Abcam, USA) at room temperature for 30 min. Color reaction was developed by DAB buffer. PBS was used as negative control.

Under light microscope examination, LMP1 and Cripto-1 positive cells were identified as the presence of yellowish to brownish stain in the membrane and cytoplasm. Based on the intensity of the color, the expression levels were classified as: 0 (negative), 1 (weak positive), 2 (moderate positive) and 3 (strong positive). Using a double-blinded scoring approach, we randomly selected 5 fields and 200 cells in each field. Based on the number of positive cells counted, positive levels were classified as: 1 (0–10% positive cells), 2 (11–50% positive cells), 3 (51–80% positive cells) and 4 (81–100% positive cells). The overall LMP1 and Cripto-1 protein expression levels were calculated by multiplying the expression level and the positive level: 0–1 (−), 2–3 (+), 4–6 (++) and 8–16 (+++).^28^

### Cell lines and cell culture

Two cells lines were used in this study: CNE1 – human NPC cells with high differentiation and CNE1-LMP1 – CNE1 cells with stable transfection of LMP1gene. Both were purchased from the Medical College. Cells were cultured routinely in DMEM medium containing 10% fetal bovine serum (FBS, Gibco Life Technologies) at 37 °C and 5% CO_2_.

### CCK-8 cell proliferation experiment

To compare the growth rate of two cell lines, logarithmic growth phase cells were seeded to 96 well plates at the initial density of 2 × 10^3^ cells/well and subjected for viability assay using CCK-8 kit (Dojindo Laboratory) at 1, 2, 3, 4 and 5 days, respectively. The cell density was measured at 450 nm on an Enzyme linked immuno analyzer (TECAN, Sunrise).

### Verification of LMP1 expression

To verify the expression of LMP1 gene in CNE1-LMP1 and CNE1 cells, total RNA was extracted from cells by TRIzol reagent (Invitrogen, Carlsbad, CA) then reverse transcription to cDNA. All primers were synthesized by Shanghai SanGon Biotechnology Co. and that included LMP1 gene forward: 5′-CAACAACGGCAAGACTCCC-3′, reverse 5′-CCTCAAAGAAGCCACCCTC-3′ and primer size was 146 bp. Reference gene GAPDH forward: 5′-GAAGGTGAAGGTCGGAGTC-3′, reverse5′-GCTCCTGGAAGATGGTGATG-3′ and primer size was 233 bp. The volume of PCR reaction system was 20 μL (SYBR Green I nucleic acid dye 10 μL, the forward and reverse primers each 0.4 μL, template cDNA 2.0 μL, and diethyl pyrocarbonate water for 7.2 μL). PCR of 40 cycles was carried out under the following conditions: 95 C for 30 s, 95 °C for 5 s, 60 °C for 20 s. Amplified product detected by agarose gel electrophoresis to verify the size of the strip and ensure the specificity of PCR products. The ΔΔCq method was utilized for relative quantification, ΔΔCt = experimental group (Ct target gene − Ct GAPDH) − control group (Ct target gene − Ct GAPDH).

### Examination of Cripto-1 expression

To examine the expression of Cripto-1 gene in CNE1-LMP1 and CNE1 cells, RT-PCR was carried out as described above using Cripto-1 gene forward: 5′-CCCAAGAAGTGTTCCCTGTG-3′, reverse 5′-TGCAGACGGTGGTAGTTCTG-3′, and primer size was 138 bp.

### Western blot assay of LMP1 and Cripto-1 proteins

The proteins were extracted from logarithmic growth cells from the culture flask and protein concentration was determined by BCA method. For the Western blot assay, the loading quantity for LMP1 protein and Cripto-1 protein were 80 μg and 30 μg, respectively. Subsequently, each protein sample was separated using 12% SDS-PAGE gel electrophoresis prior to be transferred onto PVDF membrane. The membranes were blocked at room temperature for 1 h with TBST containing 5% skimmed milk prior to being incubated with mouse monoclonal (CS 1–4, Abcam) to EBV LMP 1 (1:1000) (ab-78113, Abcam), rabbit polyclonal to Cripto-1 (1:1000) (ab19917, Abcam), rabbit polyclonal to beta Tubulin (1:500) (ab6046, Abcam) primary antibodies at 4 °C overnight, respectively. After washing with TBST solution, the PVDF membranes were incubated with corresponding second antibody (goat polyclonal anti-rabbit/-mouse IgG – H&L (1:5000) (ab6721 and ab6789, Abcam) at room temperature for 1 h. ECL liquid developed and exposed, Image J was used to analyze the gray value of each band.

### Statistical analyses

Quantitative values were expressed as means ± SD. LMP1 and Cripto-1 expression levels in various clinical pathological parameters were calculated with Chi-square test. The correlation of LMP1 and Cripto-1 expression were calculated with Spearman's Rank Correlation analysis. Comparison of the mRNA and protein expression levels between CNE1 and CNE1-LMP1 control was made with paired-samples *t*-test in all cases. All statistical analyses were performed using SPSS 21.0 with *α* = 0.05 (2 Sided) for the standard test; *p*-value less than 0.05 was considered to be statistically significant.

## Results

### Patient demographic data

The NPC group included 29 males and 13 females (*M*/*F* ratio = 2.23). Their ages ranged from 25 to 74 years (average age 52.7 ± 15.7 years old). The nasopharyngitis group included 16 males and 6 females (M/F ratio = 2.67). Their ages ranged from 20 to 59 years (average age 39.3 ± 11.4 years old). For the NPC Group, a total of 30 patients (71.4%) had neck lymph node metastasis. Histological classification showed 10 cases of keratinizing squamous cell carcinoma (23.8%) and 32 cases of non-keratotic carcinoma (76.2%). Among them, 5 cases of Stage I, 9 cases of Stage II, 18 cases of Stage III and 10 cases of Stage IV existed. The histological examination of tissue specimens obtained from nasopharyngitis patients confirmed inflammation with no sign of carcinoma.

### LMP1 protein expression in nasopharyngeal carcinoma

**Figure 1:**
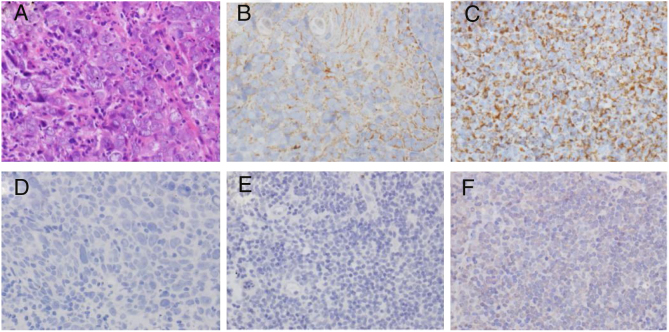
Immunohistochemical examination of LMP1 expression. (A) H&E staining of NPC tissue. (B) LMP1 expression in NPC tissue (+). (C) LMP1 expression in NPC tissue (+++). (D) Negative control of LMP1 staining in NPC tissue. (E) Negative control of LMP1 staining in nasopharyngitis tissue. (F) Weak LMP1 expression in nasopharyngitis tissue. Magnification ×400.

Examination of the localization and expression levels of LMP1 protein in nasopharyngeal mucosa specimens showed that the LMP1 protein expression rate in nasopharyngeal carcinoma was 73.8% and that in nasopharyngitis tissue was 22.7% (*p* < 0.01), respectively (Table 1). LMP1 protein distribution in nasopharyngeal carcinoma was more uniform than that in nasopharyngitis tissue (Fig. 1). Nevertheless, different LMP1 protein expression levels were seen in cancerous cells.

**Table 1:** Expression of LMP1 in nasopharyngeal carcinoma and nasopharyngitis (Chi-square test).

Group	Number	Expression of LMP1		*p*
		Positive (%)	Negative (%)	
NPC	42	31 (73.8)	11 (26.2)	
Nasopharyngitis	22	5 (22.7)	17 (77.3)	0.000

There was a statistically significant difference (*p* < 0.01) between LMP1 protein expression in patients who had lymph node metastasis (86.7%) and those without (41.7%). Further analysis of the relationship between LMP1 protein expression and NPC pathological parameters showed that the expression of LMP1 protein correlated well with TNM staging (Table 2). The expression in Stage III and IV was significantly higher than that in Stage I and II (*p* < 0.05). However, there was no statistically significant difference in terms of patient gender (*p* = 0.713), age (*p* = 0.158) and pathological type of nasopharyngeal carcinoma (*p* = 0.410).

**Table 2:** The relationship between LMP1 expression and clinical pathological parameters (Chi-square test).

Clinical parameter	Number	LMP1 expression		*p*
		Positive (%) *n* = 31	Negative (%) *n* = 11	
*Sex*				0.713
Male	29	22 (75.9)	7 (24.1)	
Female	13	9 (69.2)	4 (30.8)	

*Age*^a^				0.158
≥50 years old	26	17 (65.4)	9 (34.6)	
<50 years old	16	14 (87.5)	2 (12.5)	

*Pathological type*				0.410
Keratinizing squamous cell carcinoma	10	6 (60.0)	4 (40.0)	
Non-keratinizing carcinoma	32	25 (78.1)	7 (21.9)	

*TNM staging*				0.024^b^
I/II stage	14	7 (50.0)	7 (50.0)	
III/IV stage	28	24 (85.7)	4 (14.3)	

*Lymph node metastasis*				0.006^b^
With	30	26 (86.7)	4 (13.3)	
Without	12	5 (41.7)	7(58.3)	

a Group by median age.

b Statistical significant.

### Cripto-1 protein expression in nasopharyngeal carcinoma

**Figure 2:**
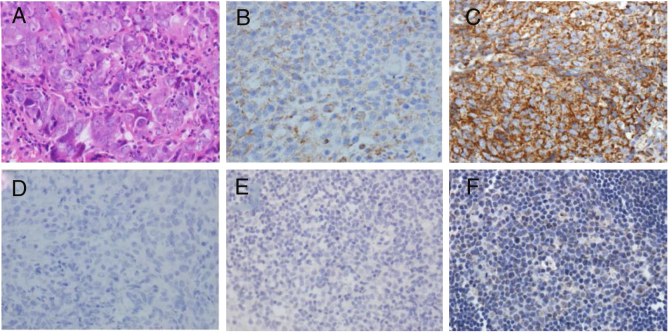
Immunohistochemical examination of Cripto-1expression. (A) H&E staining of NPC tissue. (B) Cripto-1expression in NPC tissue (+). (C) Cripto-1expression in NPC tissue (+++). (D) Negative control of Cripto-1 staining in NPC tissue. (E) Negative control of Cripto-1 staining in nasopharyngitis tissue. (F) Weak Cripto-1 expression in nasopharyngitis tissue. Magnification ×400.

Examination of the localization and expression levels of Cripto-1 protein in nasopharyngeal mucosa specimens showed that Cripto-1 protein expression rate in nasopharyngeal carcinoma was 80.9% and that in nasopharyngitis tissue was 36.4% (*p* < 0.01), respectively (Table 3). Nevertheless, different Cripto-1 proteinexpression levels were seen in cancerous cells (Fig. 2). There was a statistically significant difference (*p* < 0.01) between Cripto-1 protein expression in patients who had lymph node metastasis (93.3%) and those without (50%). Further analysis of the relationship between Cripto-1 protein expression and NPC pathological parameters showed that the expression of Cripto-1 protein correlated well with TNM staging. The expression in Stage III and IV was significantly higher than that in Stage I and II (*p* < 0.05) (Table 4). However, there was no statistically significant difference in terms of patient gender (*p* = 0.226), age (*p* = 0.688) and pathological type of nasopharyngeal carcinoma (*p* = 0.369).

**Table 3:** Expression of Cripto-1 in nasopharyngeal carcinoma and nasopharyngitis (Chi-square test).

Group	Number	Cripto-1 expression		*p*
		Positive (%)	Negative (%)	
NPC	42	34 (80.9)	8 (19.1)	
Nasopharyngitis	22	8 (36.4)	14 (63.6)	0.000

**Table 4:** The relationship between Cripto-1 expression and clinical pathological parameters (Chi-square test).

Clinical parameter	Number	Cripto-1 expression		*p*
		Positive (%) *n* = 34	Negative (%) *n* = 8	
*Sex*				
Male	29	25 (86.2)	4 (13.8)	0.226
Female	13	9 (69.2)	4 (30.8)	

*Age*^a^				
≥50 years old	26	20 (76.9)	6 (23.1)	0.688
<50 years old	16	14 (87.5)	2 (12.5)	

*Pathological type*				
Keratinizing squamous cell carcinoma	10	7 (70.0)	3 (30.0)	0.369
Non-keratinizing carcinoma	32	27 (84.4)	5 (15.6)	

*TNM staging*				
I/II stage	14	8 (57.1)	6 (42.9)	0.010^b^
III/IV stage	28	26 (92.9)	2 (7.1)	

*Lymph node metastasis*				
With	30	28 (93.3)	2 (6.7)	0.004^b^
Without	12	6 (50.0)	6 (50.0)	

a Group by median age.

b Statistical significant.

### Correlation between LMP1 and Cripto-1 expression

**Figure 3:**
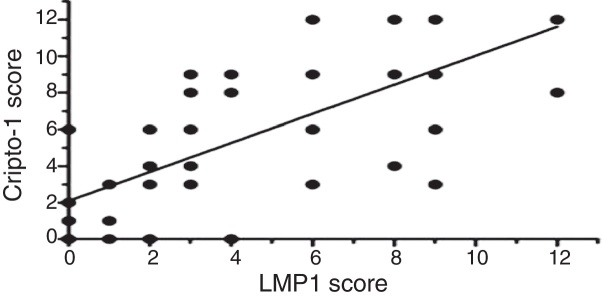
The correlation between the expression levels of LMP1 and Cripto-1.

The LMP1 protein and Cripto-1 protein expression levels obtained from immunohistochemistry staining were scored and results were listed in Table 5. The correlation assay suggested that there was a positive correlation between the expression level of LMP1 protein and Cripto-1 protein in NPC tissue (*r* = 0.758, *p* < 0.01) (Fig. 3).

**Table 5:** Correlation between the expression of LMP1 and Cripto-1 (Spearman's Rank Correlation analysis).

Expression level	LMP1				*r*	*p*
	−	+	++	+++		
*Cripto-1*						
−	6	1	1	0		
+	4	2	1	1		
++	1	5	1	2		
+++	0	2	4	11	0.758	0.000

### CNE1-LMP1 cells grown faster than CNE1 cells

**Figure 4:**
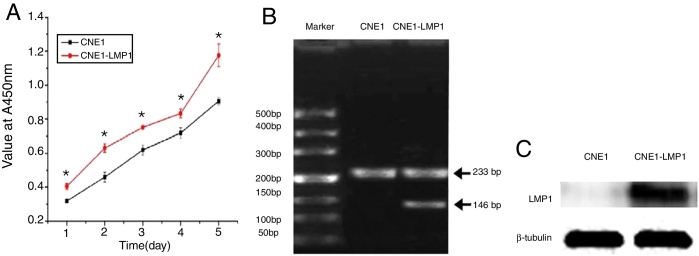
Cell growth curve of CNE1 and CNE1-LMP1 cells (*indicate *p* < 0.01) (A). The strong expression of LMP1 mRNA (B) and LMP1 protein (C) in CNE1 cells and CNE1-LMP1 cells.

Viability assay of NPC cells showed that under the same culture conditions CNE1-LMP1 cells grow faster than CNE1 cells (Fig. 4A). This might suggest that LMP1 could facilitate cell growth.

### Strong expression of LMP1 mRNA and protein in CNE1-LMP1 cells

RT-PCT assay showed that there was no expression of LMP1 Mrna (146 bp) in CNE1 cells, whereas strong expression of LMP1 mRNA was found in CNE1-LMP1 cells (Fig. 4B). Western blot assay also showed that the expression of LMP1 protein in CNE1 cells was negative but with positive expression in CNE1-LMP1 cells (Fig. 4C). Therefore, it could be confirmed that the CNE1-LMP1 cell line was stably transferred with LMP1 gene and could produce LMP1 protein.

### Strong expression of Cripto-1 mRNA and protein in CNE1-LMP1 cells

**Figure 5:**
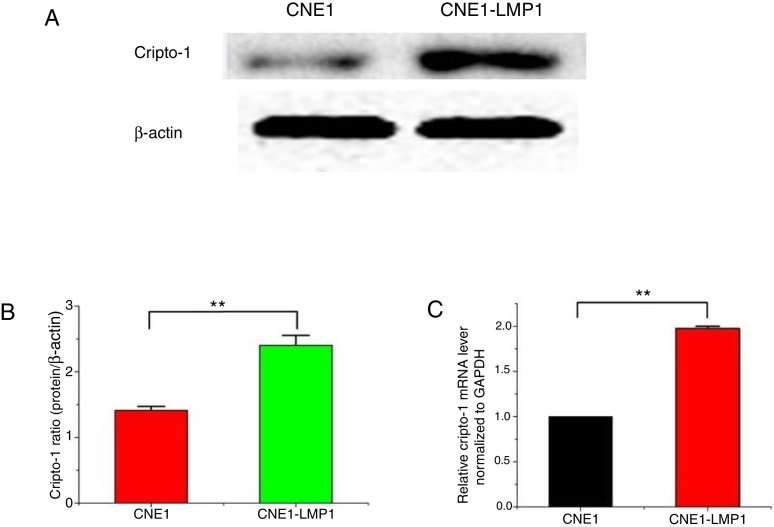
(A) The expression of Cripto-1 protein in CNE1 and CNE1-LMP1 cells and (B) quantitative analysis (*p* < 0.05). (C) The expression of Cripto-1 mRNA in CNE1 and CNE1-LMP1 cells (*p* < 0.05).

Western blot assay showed that Cripto-1 protein was expressed in both CNE1 cells and CNE1-LMP1 cells but there was significant difference in term of expression level (Fig. 5A). The quantitative assay showed that the Cripto-1 protein expression in CNE1-LMP1 cells was 1.7 times higher than that in CNE1 cells (*p* < 0.05) (Fig. 5B). Furthermore, RT-PCT assay showed that Cripto-1 mRNA was expressed in both CNE1 cells and CNE1-LMP1 cells, but there was significant difference in term of expression level. The Cripto-1 mRNA level in CNE1-LMP1 cells was 2 times higher than that in CNE1 cells (*p* < 0.05) (Fig. 5C).

## Discussion

In this study, for the first time the co-expression of LMP1 protein and Cripto-1 protein in human NPC was studied. The demonstration of co-expression of LMP1 protein and Cripto-1 protein in both NPC tissue and NPC cells suggests that such co-expression is involved in the occurrence and metastasis of human NPC.

LMP1, which is thought to be the main oncogenic protein for EBV and positively correlated with the metastasis of NPC.^29^ LMP1 can recruit a series of cytokines and interact with them to activate some key cell signaling pathways and cause apoptosis inhibition, cell proliferation, metastasis and lead to tumor genesis and development. Therefore, LMP1 can be used as a diagnostic marker of NPC.^30^, ^31^ Our study also supports this. As indicated in the *ex vivo* study, LMP1 protein was expressed in much higher rates in human NPC tissue than in nasopharygitis tissue (73.8% vs. 22.7%) (Table 1). Moreover, LMP1 over expression was associated with N classification, distant metastasis, and clinical stage (Table 2).

LMP1 can facilitate the invasiveness of EBV-positive NPC through the regulation of miR-204 expression.^32^ Invasion and metastasis are two defining characteristics in the pathogenesis and progression of malignant tumors. Metastasis is usually composed of multiple, sequential, selective and interdependent steps. The early lymph node metastasis is more common in NPC that might be driven by LMP1 from EBV. This is particular significant in immunocompetent hosts.^33^, ^34^ Indeed, in this study, *in vitro* comparison showed that CNE1-LMP1 cells exhibited higher proliferation ability than LMP1 expression negative CNE1 cells (Fig. 4A). This might be used as an indicator for screening high risk population.

Cripto-1 plays an important role in the process of embryonic development and tumorigenesis. It can initiate or promote the development of various types of cancer.^35^ In this study, an immunohistochemistry method was employed to detect the expression of Cripto-1 protein in NPC tissues. It was expressed in much higher rates in human NPC tissue than in nasopharygitis tissue (80.9% vs. 36.4%) (Table 3). This is consistent with the expression levels of Cripto-1 protein in breast cancer (i.e., 40–80%), invasive cervical cancer (65%), gastric cancer (54%) and intraductal papillary mucinous neoplasms of the pancreas (59.5%).^36^, ^37^

The correlation between Cripto-1 expression and proliferation activity or invasion/metastatic potential of cancer cells in tissue samples has been examined in *in vitro* and *in vivo* studies. Cripto-1 transduced mammary epithelial cells exhibit increased proliferation rates. In contrast, knockout of the Cripto-1 gene in mammary epithelial cells reduced the growth of these cells both *in vitro* and *in vivo*. In addition, the overexpression of Cripto-1 promoted cell proliferation, anchorage-independent growth, and transformation of mammary epithelial cells *in vitro* and induction of mammary gland hyperplasia and tumor formation in mouse mammary tumor Cripto-1 model.^38^ “knocked down the expression” of Cripto-1 in CNE-2 and C666-1 by lentivirus-mediated RNAi silencing found that the cell growth was suppressed after the inhibition of endogenous Cripto-1 protein using Cripto-1 gene silencing, the xenotransplant nude mice model with whole-body visualizing instrument found that the average volume and weight of tumor in plant mice group were significantly lower *in vivo*.^18^

In this study, an immunohistochemistry method was employed to detect the co-expression of LMP1 protein and Cripto-1 protein in NPC tissues. The results showed that both LMP1 and Cripto-1 were positive expressed in NPC, whereas inflammation epithelial tissues showed low and weak expression (Table 1, Table 2, Table 3, Table 4; Figure 1, Figure 2). In the current study, for the first time, it was demonstrated both *in vitro* and *in vivo* that LMP1 and Cripto-1 expression are positively correlated in NPC (*p* < 0.01) (Table 5; Fig. 3). Invasion, metastasis and recurrence are the basic biological characteristics that can affect the survival of NPC patients. To date, tumor metastasis often seen in cases of NPC is still the biggest obstacle to curing patients. Our results showed that LMP1 and Cripto-1 protein expression in NPC patients with neck lymph node metastasis was significantly higher than that in patients without lymph node metastasis (*p* < 0.05). This indicates that the high expression of LMP1 and Cripto-1 may play a key role in promoting NPC invasion and metastasis, which are related to the adverse progress of NPC. Compared with other head and neck cancers, the lack of typical early clinical features, high local recurrence rate and neck lymph node metastasis are major factors for poor prognosis of NPC.^39^ In many classic prognostic indicators of NPC, neck lymph node metastasis is the most important, therefore, LMP1 and Cripto-1 over expression are likely involved in the development of NPC. Based on the fact that LMP1 and Cripto-1 proteins were co-expressed in NPC and NPC cells (Figure 4, Figure 5), it is reasonable to believe that they are involved in a variety of signaling pathways in tumor development. They could have a common signal pathway effect factor. It may be a potential new target for early diagnosis and target therapy as well as a new prognostic molecular marker of NPC.

## Conclusion

As suggested by this study that LMP1 and Cripto-1 can be co-expressed in NPC. The expression of Cripto-1 is likely regulated and affected by LMP1 protein. Cripto-1 protein might be a key effector protein in signaling pathways regulated by LMP1 protein, thereby promoting tumor invasion and metastasis. Therefore, it is worth studying the effects of LMP1 regulation on the expression of Cripto-1 in invasion and metastasis of NPC to understand the molecular characteristics of NPC progression and insight into the molecular mechanism of NPC prognosis.

## Conflicts of interest

The authors declare no conflicts of interest.
